# Replication stress as a driver of cellular senescence and aging

**DOI:** 10.1038/s42003-024-06263-w

**Published:** 2024-05-22

**Authors:** Lauren M. Herr, Ethan D. Schaffer, Kathleen F. Fuchs, Arindam Datta, Robert M. Brosh

**Affiliations:** 1 Helicases and Genomic Integrity Section, Translational Gerontology Branch, National Institute on Aging, National Institutes of Health, Baltimore, MD USA; 2 Department of Cancer Biology, Perelman School of Medicine, University of Pennsylvania, Philadelphia, PA USA

**Keywords:** Stalled forks, Cancer

## Abstract

Replication stress refers to slowing or stalling of replication fork progression during DNA synthesis that disrupts faithful copying of the genome. While long considered a nexus for DNA damage, the role of replication stress in aging is under-appreciated. The consequential role of replication stress in promotion of organismal aging phenotypes is evidenced by an extensive list of hereditary accelerated aging disorders marked by molecular defects in factors that promote replication fork progression and operate uniquely in the replication stress response. Additionally, recent studies have revealed cellular pathways and phenotypes elicited by replication stress that align with designated hallmarks of aging. Here we review recent advances demonstrating the role of replication stress as an ultimate driver of cellular senescence and aging. We discuss clinical implications of the intriguing links between cellular senescence and aging including application of senotherapeutic approaches in the context of replication stress.

## Abstract (web summary)

This review discusses recent advances demonstrating the role of replication stress as an ultimate driver of cellular senescence and aging, highlighting connections to hallmarks of aging, hereditary accelerated aging disorders, and senotherapeutics.

## Introduction

Replication stress can be caused by an endogenous or environmental condition that disrupts the faithful copying of the genome (Table 1)^1^. In addition to metabolites, drugs, and radiation that can introduce nuclear or mitochondrial genomic DNA damage and interfere with replication-associated processes, genetic deficiencies can perturb replicative DNA synthesis or fork stability. Replication fork stalling and demise contribute profoundly to genomic DNA damage and cellular senescence characterized by irreversible replication arrest^2^. Understanding the molecular-genetic defects and environmental causes of replication stress is paramount to characterizing the mechanism of aging, determining biomarkers for aging, and developing new treatment strategies and cures for age-related diseases. Cells of neoplastic tissues experiencing replication stress must be refreshed with nutrient macromolecules so they can perform vital functions. Alternatively, to suppress aging, senescent cells are cleared and replaced with newly divided and differentiated cells. Thus, replication stress as a driver of cellular senescence and aging is critically important to characterize.

**Table 1:** Endogenous and exogenous agents of replication stress^a,b^

Genomic DNA Structure	Cellular Condition
Endogenous Alternate DNA Structures	DNA-RNA hybrids (R-loops)^120,193^ G-quadruplex DNA (stabilized by G4 ligand)^194–198^ I-motif DNA (stabilized by I-motif ligand)^197,199^ Telomere-specific DNA conformation (e.g., T-loop)^200,201^ Reversed fork caused by reactive oxygen species (can be induced by replication inhibitor drug)^202^
DNA Damage Induced by Endogenous Biochemical Processes	DNA interstrand cross-links induced by reactive formaldehydes^203^ DNA-protein cross-links (can also be exogenously induced)^204–206^
DNA Damage Induced by Radiation and Environmental Agents	Bulky DNA adducts (e.g., pyrimidine dimers) induced by ultraviolet light^207,208^ Bulky DNA adducts induced by environmental DNA-damaging chemicals (e.g., polycyclic aromatic hydrocarbons)^209,210^ DNA interstrand cross-links induced by exogenous agents (e.g., psoralen)^211^
DNA Damage Induced by Chemotherapy Drugs	Bulky DNA adducts (e.g., alkylated bases) induced by selected chemotherapeutic drugs^212,213^
Replication Inhibition Caused by Chemotherapy Drugs	Drugs that cause nucleotide depletion (e.g., hydroxyurea)^214,215^ Topoisomerase-binding compounds causing torsional stress (e.g., low dose camptothecin)^69,216^ DNA polymerase inhibitors^217^

^a^The table is not meant to be exhaustive, but rather illustrate the wide range of endogenous or exogenously induced sources of replication stress for mammalian cells.

^b^Replication stress by genetic manipulation of DNA replication factors, etc. via RNA interference or CRISPR technology is not included in the table, but some examples are mentioned in text.

The dire consequences of a poor response to replication stress are clinically apparent in genetically inherited premature aging disorders^3^. A common feature of these diseases is chromosomal instability, prompting an investigation of the cellular pathways responsible for a compromised response to replication stress. Endogenous or exogenously induced DNA damage derails normal replicative DNA synthesis and contributes to aging at the cellular and tissue-specific levels.

Although replication stress has long been considered a nexus for DNA damage, its role in aging is under-appreciated. We describe phenotypes and pathways evoked by replication stress that align with hallmarks of aging. Furthermore, many age-related diseases are characterized by compromised replicative DNA synthesis and chromosomal instability, sparking renewed interest in the search for biomarkers of aging and potential targets to ameliorate phenotypes associated with replication stress that might translate to clinical therapies (e.g., senotherapeutics^4^).

Replication stress contributes to the dysregulation of many hallmarks of aging, which are extensive^5^. Difficulty in replicating chromosome ends causes telomere attrition or telomeric DNA damage that perturbs binding of the protective shelterin complex, commonly observed during cellular senescence^6^. Depletion of the stem cell (SC) reservoir by replication stress promotes decline of tissues and organs in a cell lineage-dependent manner^7^. For example, replication stress drives functional loss in old hematopoietic SCs^8^. Inducible double-strand breaks (DSBs) caused mouse liver aging^9^; these highly toxic lesions are associated with cellular senescence and apoptosis^10^ and potentially trigger the senescence-associated secretory phenotype (SASP) that mediates intercellular signaling and is a representative feature of aging cells^11^. Moreover, even a low level of inducible DSBs in a transgenic mouse model was found to cause epigenetic changes and dysfunctional transcription networks associated with age-related phenotypes^12^. Genetic backgrounds or cellular conditions conducive to perturbed replicative DNA synthesis display abnormal epigenetic regulation which affects transcription and histone recycling^13^. Moreover, retrotransposable elements that alter chromatin state can lead to a sustained inflammatory response^14,15^.

Replication stress in mitochondria (mt) compromises their function^16^, which is interconnected with other aging hallmarks that underlie poor energy production and frailty^17^. Cytosolic nucleic acids including DNA fragments from mitochondria or nuclei are detected by specialized sensors which trigger proinflammatory cytokines that induce an innate immune response by the cGAS-STING pathway^18^, resulting in senescence and compromised SC function. Altogether, nearly all hallmarks of aging are elicited by a poor response to replication stress.

In this review, we will discuss these topics to frame the case that replication stress represents a driving force for cellular senescence and aging phenotypes.

## Hereditary diseases characterized by cellular phenotypes characteristic of a defective response to replication stress

There continues to be growing interest in rare hereditary diseases characterized by an accumulation of genomic DNA damage and accelerated aging that provide insights into normal aging^19^. Many genetic disorders characterized by clinical symptoms of premature aging display cellular phenotypes that also indicate a condition of heightened replication stress (Table 2). These hereditary diseases provide a window of opportunity to better understand pathways of genome metabolism responsible for chromosomal stability and underlying mechanisms in which replication stress is driving not only cellular senescence but also the accelerated decline of bodily function, including (but not exclusively) replicative tissues characterized by persistent inflammatory processes. Some salient and notable points are discussed in this section.

**Table 2:** Hereditary accelerated aging disorders characterized by heightened replication stress^a,b^

Genetic Disorder	Mutated Gene	Prominent Clinical Features	Replication Stress Cellular Phenotypes
Ataxia Telangiectasia	*ATM*^218^	Cerebellar ataxia Progressive neuromotor deterioration Immunodeficiency Atrophy and hyperpigmentation	Defective coupling of replication stress response to metabolic remodeling during cellular senescence^219^ Impaired mobilization of signaling response to DSBs; for review, see^220^ In yeast, ATM supports replisome stability during replication stress^221^
Bloom Syndrome	*BLM*^222^	Growth deficiency Sun-sensitivity High cancer risk Abnormal immune responses	Reduced rate of DNA synthesis and fork movement^223,224^ Abnormal profile of DNA replication intermediates^225^ Reduced replication fork progression after hydroxyurea exposure^226^ Hypersensitivity to hydroxyurea^227,228^ Defective processing of late-replicating DNA intermediates^229^
Cockayne Syndrome	*CSA*^230^, *CSB*^231^	Severe photosensitivity Impairment of physical development Progressive neurological degeneration Cataracts Hearing loss	Hypersensitivity to agents that induce replication stress; for review, see^232^ CSB-deficient cells exposed to hydroxyurea display slowed fork progression^51^ CSB regulates fork degradation in BRCA1- or BRCA2-deficient cells^51^ CSB-deficient cells are compromised in mitotic DNA synthesis^233^
Dyskeratosis Congenita, Hoyeraal-Hreidarsson Syndrome	*DKC1, RTEL1, DCLRE1B, NHP2, NOP10, NPM1, PARN, RPA1, TERC, TERT, TINF2, WRAP53, CTC1* *For review, see*^42^	Bone marrow failure Oral leukoplakia Reticular skin pigmentation Abnormal nail formation	Generalized shortened telomeres in affected patients; for review, see^42,234,235^ RTEL1-depleted cells accumulate R-loops at sites of active replication^236^ HHS-linked RTEL1 mutations cause replication defects in unstressed cells^237^ RPA suppresses G4 formation, allowing proper telomere maintenance^238^ STN1 facilitates concerted G- and C-strand synthesis to regulate telomere length^239^ TIN2 helps to prevent ATR signaling during telomere replication and repress sister telomere association^240^ CTC1 in CST complex aids in C-strand fill-in DNA synthesis; also, genome-wide role to restart stalled forks; for review, see^234^
Fanconi Anemia	*FANC-A, B, C, D1, D2, E, F, G, I, J, L, M, N, O, P, Q, R, S, T, U, V, W* *For review, see*^36^	Progressive bone marrow failure Abnormalities in digits and stature Predisposition to cancer Malformation of organs	FANCD2 depletion compromises resolution of under-replicated DNA in SETX-deficient cells^121^ FANCJ-deficient cells exposed to G4-stabilizing ligands display reduced replication rate; for review, see^86^ FA-mutated cells exposed to DNA cross-linking drugs are deficient in replication restart by BIR pathway^241^ FA-deficient cells display destabilized replication forks stressed by DNA damage; for review, see^149^
Hutchinson-Gilford Syndrome	*LMNA*^242^	Loss of subcutaneous fat Alopecia Failure to thrive during infancy Hearing, sight, and dental loss Disproportionate facial features	Replication fork stalling and induction of an interferon-like response^45^ Lmna-/- MEFS exhibit impaired telomere maintenance^243^ HGPS cells display telomere attrition^244^ Defective replication fork restart after hydroxyurea exposure^245^
RECON Syndrome	*RECQL1*^68^	Skeletal and joint abnormalities Photosensitivity Progeroid appearance Xeroderma	Reduced replication in presence of topoisomerase inhibitors^68^ Reduced ability to restart forks stalled by methylmethanesulfonate or hydroxyurea^68^
Ruijs-Aalfs Syndrome	*SPRTN*^53^	Lipodystrophy Muscular atrophy Low body weight Early onset hepatocellular carcinoma Neoplasms	Reduced DNA replication rate in SPRTN-knockout cells^246^ SPRTN-knockout cells accumulate DSBs in S-phase^246^ SPRTN facilitates replication bypass of formaldehyde-induced DNA-protein cross-links^56^
Seckel Syndrome	*ATR*^247^	Severe dwarfism Craniofacial features Microcephaly Intellectual disability	Seckel patient cells display impaired DNA damage response^247^ ATR-Seckel model is characterized by replication stress and accelerated aging^248^ Chromosomal breakage at fragile sites in Seckel syndrome cells after aphidicolin exposure^249^
Warsaw Breakage Syndrome	*DDX11*^250^	Microcephaly Pre- and post-natal growth retardation Abnormal skin pigmentation Cochlear anomalies; hearing loss	Reduced replication fork progression after hydroxyurea exposure^59^ Impaired binding of cohesin to chromatin during S-phase^251^ Reduced replication fork speed after G4 ligand exposure^62^
Werner Syndrome	*WRN*^252^	Bilateral ocular cataracts Greying and loss of hair Scleroderma appearance of skin Short stature Pinched facial features	Prolonged S-phase and reduced initiation of DNA synthesis^253–255^ Reduced replication fork progression and recovery after DNA damage or fork stalling^226,256^ Defective telomere lagging strand DNA synthesis^24^ Sensitivity of WRN-depleted cells to hydroxyurea^228^

^a^The genetic diseases listed in the table are characterized by some but not all features of premature aging.

^b^Representative cellular phenotypes characteristic of replication stress due to a deficiency in the gene mutated in the disease are listed.

### RECQ genetic disorders caused by deficiencies in helicases with unique but also overlapping functions

Of the five conserved human RECQ helicase genes, mutations in four (*WRN, BLM, RECQL4, RECQL1*) are linked to distinct hereditary premature aging disorders all characterized by genomic instability and a cellular phenotype of compromised response to replication stress (Table 2)^20,21^. While the RECQ helicases all share a conserved ATPase/helicase core domain and some overlap in their catalytic functions, nucleic acid substrate specificity, and protein interactions^22^, the growing consensus is that each RECQ has unique roles in pathways of genome metabolism that presumably reflect the different clinical features of each genetic disease; however, these relationships are complex. For example, studies of human cells and mouse models have demonstrated important roles of both the WRN^23–25^ and BLM^25–28^ helicases in telomere metabolism, suggesting that these two RECQs are specially tailored during cellular DNA replication to resolve unusual nucleic acid structures prone to occur at chromosome ends, as well as more broadly in the genome (Table 2).

Consistent with their unique nature, there are distinct genetic interactions of the five mammalian RECQ helicases^29^, a topic of great interest in cancer biology given their up-regulation in various cancers^20^ and potential as anti-cancer drug targets^30^. For example, WRN has been implicated as an essential factor for survival and proliferative capacity of tumors characterized by microsatellite instability (for review, see ref. ^31^). The discovery of helicase inhibitors such as one in a clinical trial (Study Details | Study of HRO761 Alone or in Combination in Cancer Patients With Specific DNA Alterations Called Microsatellite Instability or Mismatch Repair Deficiency. | ClinicalTrials.gov), and their applicability in vitro with human cancer cell lines^32–34^ and in vivo with a *BRCA2* xenograft mouse model^35^ suggest that continued pursuit of RECQ pharmacological modulation to combat cancer in preclinical models may translate to efficacious strategies in the clinic.

### Bone marrow failure diseases Fanconi anemia and dyskeratosis congenita

Characterized as a bone marrow failure disorder, Fanconi Anemia (FA) stems from mutations in >20 genes that play an integral role in the cellular response to DNA damage and replication stress^36^ (Table 2). While originally thought to be strictly responsible for repair of DNA interstrand cross-links (ICLs), the FA pathway with direct involvement of *BRCA* genes is implicated in DSB repair by homologous recombination (HR) and break-induced replication at collapsed or reconstructed replication forks. For many years, it was suggested that lipid peroxidation was the primary source of endogenous ICLs susceptible to repair by the FA pathway^37^; however, more recent evidence implicates the accumulation of formaldehyde which reacts with macromolecules, including DNA, lipids, and proteins, to be a major culprit^38^. Precisely how ICLs affect replication fork-associated events is still a major area of investigation, but it is evident that formaldehyde-induced DNA damage causes replication stress and the *FA/BRCA* genes play a significant role in its alleviation^39–41^. As discussed below, replication stress drives the decline of hematopoietic SCs (HSCs), a characteristic feature of FA, and may more broadly and adversely affect SC compartments implicated in aging of tissues and organs beyond the hematopoietic lineage.

Like FA, Dyskeratosis Congenita (DC) and the related, more severe disease Hoyeraal-Hreidarsson Syndrome (HHS) are also genetically complex disorders characterized by bone marrow failure^42^. Telomere defects are implicated in DC/HHS, with evidence that telomeric DNA synthesis is compromised (Table 2). Thus, an emerging theme is that difficult-to-replicate telomeres pose a unique challenge to cells that require specialized genetically controlled pathways to maintain their stability.

### Hutchinson-Gilford progeria syndrome caused by perturbed nuclear envelope

A genetic splicing variant of the lamin A/C gene (*LMNA*) encodes an aberrant lamin A protein (progerin) that results in defective structural filamentous protein incorporated into the nuclear skeleton^43^. Transient nuclear ruptures perturb replication and induce DNA damage via changes to chromatin structure and reduced nuclear replication protein recruitment^44^. This discoordination of replication factors causes replication stress and p53-activated senescence of Hutchinson-Gilford Progeria Syndrome (HGPS) cells. The consequential elevation in DNA damage signaling activates cell cycle checkpoints that mediate reduced cell cycle progression in HGPS cells, which is believed to drive accelerated aging. Many advances have been made in understanding the mechanistic basis for HGPS phenotypes that revolve around a poor response to replication stress and telomere defects (Table 2), deriving from mechanical stress that causes problems with DNA synthesis and genomic stability that in turn drive aging processes. The Gonzalo lab and collaborators determined that cellular aging induced by progerin expression caused replication fork stalling and degradation accompanied by activation of the interferon (IFN)-like response^45^. Moreover, these characteristics could be rescued pharmacologically by cellular reprogramming agents.

### Ataxia Telangiectasia and Seckel Syndrome display defects in signaling DNA damage and replication stress

Mutations in the *ATM* and *ATR* genes which encode protein signaling kinases are linked to the premature aging disorders Ataxia Telangiectasia (AT)^46^ and Seckel Syndrome (SS)^47^, respectively. Classically, ATM and ATR were assigned checkpoint roles in response to DSBs and replication stress, respectively; however, emerging evidence suggests that they both play roles during DNA synthesis that influence chromosomal stability and cellular senescence (Table 2), which together underlie the accelerated aging features characteristic of the two disorders. AT is classified as a neurodegenerative disorder, whereas SS is a growth and developmental disease with intellectual disability apparent. It is believed that loss of checkpoint functions due to these genetic mutations causes DNA damage-induced dysfunction of somatic SCs and defective tissue maintenance that are both critical for development and normal aging.

### Cockayne syndrome

Cockayne Syndrome (CS), originally identified to be a disease defective in the preferential nucleotide excision repair of transcriptionally active genes, arises due to bi-allelic mutations in the *CSA* or *CSB* genes^48^. *CSA* encodes a protein with a tryptophan-aspartic acid (WD) repeat, a moiety that often serves as a scaffold for protein or DNA interactions involved in transcriptional regulation or chromatin remodeling. CSA is thought to mediate key interactions of proteins with RNA polymerase II stalled at sites of bulky DNA damage^49^, whereas the CSB protein is an ATP-dependent DNA translocase thought to push the stalled RNA polymerase II forward to bypass the obstacle^50^. In addition to its transcription-coupled repair mode, experimental evidence from cell-based assays places CSB as an important player in the response to replication stress (Table 2). CSB biochemically acts to reverse stalled replication fork-like structures in a manner similar to the DNA translocases SMARCAL1, ZRANB3, and HLTF^51^. In so doing, CSB acts in the same pathway as the structure-specific nuclease MRE11 to restart stalled replication forks in human cells. It remains unclear if or how CSB’s activity is orchestrated with the other DNA translocases implicated in fork reversal to regulate fork progression throughout the genome. However, it was determined that co-deficiency of CSB and SMARCAL1 synergistically promotes recruitment of the structure-specific nuclease MUS81 to telomeres in cells that operate by the alternative lengthening of telomeres (ALT) pathway dependent on HR^52^. Moreover, ALT cells co-deficient in CSB and SMARCAL1 display elevated fragile chromosomes which are believed to be a consequence of fork stalling at telomeric regions.

### Ruijs-Aalfs syndrome

The *SPRTN* gene, mutated in the premature aging disorder Ruijs-Aalfs Syndrome, encodes a PCNA-interacting protein that operates in DNA damage tolerance^53^. Phenotypic deficiency of SPRTN was first observed in transgenic mice which displayed chromosomal instability, cellular senescence, and premature aging features including cataracts, lordokyphosis (curvature of the spine), and cachexia (wasting) early in life^54^. Biochemical studies revealed that SPRTN encodes a metalloprotease that digests covalent DNA-protein complexes (DPCs) and that the clinical mutations reduced its substrate cleavage activity compared to recombinant wild-type SPRTN^55^. It remains to be fully understood how defective DPC processing gives rise to the characteristic premature aging features and predisposition to liver cancer. However, it was determined that SPRTN-deficient cells display reduced replication fork speed following subjection to formaldehyde that induces DPCs^56^ (Table 2), suggesting a role of SPRTN in facilitating replication via its DPC processing function. Subsequent research demonstrated that SPRTN proteolytically attacks the checkpoint kinase CHK1, activating CHK1’s phosphorylation of SPRTN which enhances its recruitment to chromatin to allow smooth replication and DPC repair^57^.

### Warsaw Breakage Syndrome

Warsaw Breakage Syndrome (WABS) is classified as a cohesinopathy disorder characterized by developmental abnormalities^58^. Although WABS does not fully resemble a classic premature aging disorder, the pre- and post-natal growth inhibition is accompanied by chromosomal instability, a hallmark of many more traditional hereditary accelerated aging diseases. The mutated *DDX11* gene encodes a DNA helicase that interacts with proteins involved in replication fork protection and stability^59^ (Table 2). Cells from WABS patients display reduced replication fork progression^59^. Moreover, cancer cell lines depleted of DDX11 by RNA interference or in which DDX11 was deleted by CRISPR were found to be hypersensitive to chemotherapy drugs that induce replication stress^60^. DDX11 is believed to resolve G-quadruplex (G4) DNA to enable smooth replication^61–63^. However, the precise relationship of G4 DNA metabolism to aging and the mechanistic function(s) of DDX11 in G4-induced replication stress have not yet been fully elucidated.

## Fork instability is a common feature of replicative stress

At the molecular level, accumulation of replicatively senescent cells characterizes biological aging. Replication stress induced by various exogenous and endogenous sources triggers DNA damage response (DDR) pathways that act as barriers to prevent neoplastic transformation of cells harboring damaged DNA. However, chronic DDR activation may also lead to premature replicative senescence and accumulation of senescent cells over time that contribute to human aging. Therefore, replication stress is thought to directly contribute to biological aging processes^64,65^. Replication stress is defined as stalling or slowing of replication fork progression which may lead to replication collapse and DNA damage. Stalled forks need to be protected and recovered to resume DNA synthesis and prevent genomic instability, a hallmark of aging. A direct link between compromised stalled fork recovery and aging has been established by characterizing the molecular phenotypes of cells isolated from individuals with Werner Syndrome (WS), an autosomal recessive premature aging disease resulting from loss-of-function mutations in the *WRN* gene (Table 2). Over the years, experimental evidence has demonstrated critical functions of the RECQ helicase WRN in stability and recovery of stalled replication forks under conditions of replication stress. Consistent with the roles of WRN in processing and stabilizing stalled forks, WS fibroblasts show reduced DNA replication capacity, fork asymmetry, heightened genomic instability, and premature replicative senescence. These phenotypes are likely attributed to failure to resolve complex replication intermediates resulting from stalled replication forks upon functional loss of WRN.

**Fig. 1:**
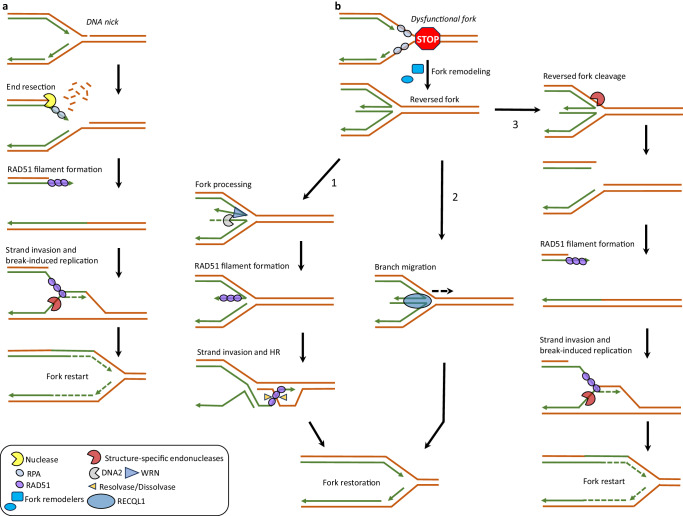
Mechanisms of stalled fork recovery under conditions of replication stress. Replication stalling or fork collapse upon exposure to various exogeneous and endogenous stressors activates DNA damage response pathways critical for stalled fork stabilization or restoration and replication restart. **a** A replication fork encountering an ssDNA break/gap can be recovered by generating a DSB, thereby allowing HR-dependent fork restoration. Nucleases involved in DNA end resection such as MRE11 or EXO1 process the break and generate a 3’ ssDNA overhang necessary to initiate HR at the broken fork. ssDNA-bound RPA is replaced by RAD51 recombinase which in turn allows for strand invasion into the sister chromatid, thereby forming a displacement (D)-loop structure to initiate break-induced replication using the sister chromatid as template. The D-loop can be cleaved by structure-specific endonucleases such as MUS81 to restore the replication fork and continue DNA synthesis. **b** A dysfunctional fork such as one stalled due to nucleotide depletion is acted upon by the coordinated actions of fork remodeling enzymes and RAD51 to generate a reversed fork intermediate. Fork protecting factors such as BRCA1, BRCA2, RAD51, WRN, and RECQL1 protect reversed forks from unscheduled pathological degradation by nucleases. However, helicase-assisted nucleolytic processing (e.g., WRN-DNA2) of stalled forks (**1**) in a controlled manner promotes replication restart through HR. Alternatively, a reversed fork can be restored via the branch-migration activity of RECQL1 helicase (**2**). The reversed fork can also be cleaved by structure-specific endonucleases (e.g., MUS81) to generate a single-ended double strand break (seDSB) at the fork that undergoes homology-directed fork restoration (**3**), as described in **a**. Cells genetically deficient in fork-stabilizing proteins (e.g., WRN helicase mutated in WS) accumulate DSBs at forks and experience gross chromosomal instability which may lead to replicative senescence and accelerated aging phenotypes.

In addition to WS, hereditary diseases linked to other RECQ helicases implicated in stalled fork processing (BLM, RECQL1) exhibit some molecular phenotypes related to premature aging (Table 2). Thus, replication fork instability resulting from the loss of critical fork maintenance proteins potentially drives the onset of replicative senescence which in turn leads to accelerated decline of function at the cellular, tissue, and organ levels. Below, we discuss how coordinated functions of various proteins implicated in DNA processing ensure replication fork stability and DNA synthesis resumption following fork stalling (Fig. 1).

### DNA helicases are key players in replication fork integrity

Cells have distinct but interconnected mechanisms that ensure protection and accurate restart of stalled replication forks. A battery of helicases and nucleases act on stalled or damaged replication forks and catalytically process replication fork intermediates. State-of-the-art cell-based techniques such as Isolation of Proteins on Nascent DNA (iPOND)^66^ or In-Situ Protein Interactions with Nascent DNA Replication Forks (SIRF)^67^ have provided direct evidence for enrichment of these enzymes specifically at forks in a replication stress-dependent manner. The RECQ class of ATP-dependent DNA helicases (RECQL1, WRN, BLM, RECQL4, and RECQL5) are fork-associated proteins that play vital roles in resolving complex replication intermediates generated during replication, recombination, and repair processes. Fork restoration activity of RECQL1, mutated in the genomic instability disorder RECON syndrome^21,68^, converts a stalled replication fork from its regressed state to an active fork, thereby promoting replication restart^69^. In addition, RECQL1 can protect stalled replication forks from DNA2 nuclease-mediated degradation^70^. Accordingly, clinically relevant *RECQL1* mutations impair its fork restoration activity in vitro as well as reduce fork restart ability and increase fork degradation as demonstrated by cell-based DNA fiber experiments^68^.

The increased spontaneous and drug-induced chromosomal aberrations (chromatin breaks and gaps) in RECON syndrome patient-derived cells underscore the importance of RECQL1-mediated fork integrity maintenance to suppress chromosomal instability. BLM and WRN are two helicases vital to rescue stalled replication forks and maintain genomic stability. BLM helicase, mutated in Bloom’s Syndrome (BS), mitigates replication stress by promoting efficient fork restart and suppressing new replication origin firing in a manner that depends on its helicase activity and genetic interaction with the FA pathway protein FANCD2^71,72^. BS patients exhibit characteristics of premature aging, and human *BLM* expressed in yeast has been shown to suppress premature aging phenotypes of *sgs1Δ* mutants^73^, suggesting that persistent replication stress serves as a driver of aging. Like BLM, WRN helicase also promotes fork restart by assisting nucleolytic processing of stalled forks by DNA2 exonuclease, thereby preventing replication fork collapse and DSB generation at forks^70^. In addition, WRN exonuclease activity protects stalled forks from unscheduled nucleolytic degradation by MRE11 nuclease, thus promoting genome stability^74^. In cooperation with RAD51, WRN also plays a structural role in counteracting unscheduled degradation of stalled forks by MRE11 nuclease^75^. Moreover, WRN helicase has been reported to promote stabilization and recovery of deprotected stalled forks in *BRCA2*-deficient cells^35,76^. Given the essential role of WRN in fork recovery, it follows that WS cells exhibit spontaneous fork stalling, increased chromosomal instability, and early senescence features.

Another important aspect of WRN-mediated genome stability maintenance is its well-characterized role in supporting replication of telomeric DNA rich in repeat sequences. This is attributed to its functions in resolving DNA topological structures such as G4s or T-loops prevalent in telomeres. Importantly, consistent with its telomere localization and role in telomere replication^24,77^, WRN deficiency results in telomeric defects coupled with premature aging phenotypes in late-generation and telomerase-deficient mice harboring ‘pre-shortened’ telomeres^23,25^. RECQL4, mutated in Rothmund–Thomson syndrome (RTS), controls replication initiation by assisting with loading of the replicative helicase^78,79^. RTS and the RECQL4-related genetic diseases Baller-Gerold Syndrome and RAPADILINO are heterogeneous disorders with a spectrum of diverse clinical features, including poikiloderma, skeletal abnormalities, and craniosynostosis^80^. Although mutation of RECQL5 has not been linked to any genetic disorders to date, it has emerged as an important player in stabilizing stalled forks during replication stress^81,82^. RECQL5 also promotes DNA repair synthesis at common fragile sites during mitosis by facilitating MUS81-EME1 endonuclease-mediated cleavage of late replication intermediates^83^. Importantly, RECQL5 suppresses replication stress arising from replication-transcription conflicts by resolving DNA replication intermediates^84^.

In addition to the RECQ helicases, FANCJ and DDX11, belonging to the family of Iron-Sulfur (Fe-S) cluster helicases^22^, play crucial roles in the replication stress response. The BRCA1-interacting FANCJ helicase implicated in the BRCA-FA pathway protects replication forks from MRE11-mediated degradation by controlling HLTF-mediated remodeling of stalled forks, thereby counteracting replication stress^85^. FANCJ is also crucial for G4 resolution that can perturb fork progression^86^. DDX11 helicase, mutated in the chromosomal instability disorder WABS, mitigates replication stress by promoting stalled fork recovery in cooperation with Timeless, a component of the replication fork protection complex^59^, and by promoting DNA end resection at damaged replication forks^60^. Importantly, experimental evidence demonstrates that FANCJ^86^ and DDX11^61–63^ helicases facilitate replication and DNA damage repair through difficult-to-replicate G-rich genome sequences. FANCM, a DNA-dependent ATPase and translocase implicated in the FA pathway, also plays a vital role in accurate repair of stalled forks. Using its conserved ATPase activity, FANCM promotes stalled fork reversal and error-free HR-mediated stalled fork repair in conjunction with BLM helicase^87–90^.

### Structure-specific nucleases and fork protection factors suppress replication stress by their action at stalled or regressed forks

When fork movement is challenged by replication stress, the forks undergo reversal to generate “four-way” reversed fork structures. This is a global response to replication stress-induced uncoupling of replicative polymerase and helicase and is primarily catalyzed by the HR factor RAD51 and vital DNA translocase enzymes such as SMARCAL1, HLTF, and ZRANB3^91^. Fork reversal is critical to stabilizing stalled forks, allowing DNA synthesis past the stall-inducing lesion by a template-switching mechanism and repair of damaged DNA by excision repair or recombination-mediated repair. Reversed fork structures are preferred substrates for nucleolytic processing by nucleases implicated in stalled fork recovery and genome maintenance. One such nuclease is DNA2, which processes reversed fork ends following replication stalling in a WRN helicase-dependent manner and creates partially resected reversed fork intermediates amenable to restart by branch migration or HR mechanisms^70^. MRE11, CtIP, and EXO1 nucleases have also been reported to perform end processing of stalled fork intermediates to promote HR-mediated fork restart^92–94^; however, the underlying mechanisms are distinct from DNA2-mediated stalled fork processing and dependent on specific genetic backgrounds^95^. An important backup mechanism of stalled fork recovery is mediated by the structure-specific endonuclease MUS81, which becomes activated as a last resort when other fork recovery mechanisms become unavailable. MUS81 cleaves stalled fork intermediates to generate DSBs at forks, thereby favoring HR-mediated replication restart^96,97^.

Although nucleolytic processing of stalled forks is necessary for replication restart, uncontrolled nucleolytic degradation of stalled replication intermediates can lead to fork collapse and instability. Fortunately, stalled forks are protected from unscheduled nucleolytic degradation by the crucial FA/HR pathway proteins BRCA1, BRCA2, FANCD2, and RAD51^95,98,99^. In genetic backgrounds deficient in major fork-protecting factors, stalled forks undergo MRE11 and EXO1 nuclease-mediated pathogenic degradation, leading to genomic instability^95,100^. Importantly, experimental evidence suggests that BRCA1/2-mediated stalled fork protection is also critical for timely restart as BRCA1/2-deficient cells exhibit delayed restart following replication stalling^35,101^. Aside from BRCA1 and BRCA2, other members of the BRCA1-A complex (RAP80, ABRAXAS, MERIT40, BRCC36, and BRCC45) have been implicated in stalled fork protection and recovery following replication stress^102–104^. Thus, principal components of HR repair play vital roles in rescuing stalled replication forks, thereby maintaining genome integrity under conditions of replication stress.

Replication stress induces changes in chromatin, and experimental evidence points toward important roles of stress-induced chromatin modifications in stalled fork protection and rescue. For example, methylation of histone H3 Lys4 (H3K4) by SETD1A methyl transferase enhances FANCD2-dependent chromatin remodeling, thereby stabilizing RAD51 nucleofilaments to prevent nucleolytic degradation of stalled forks^105^. In another study, Thakar et al. showed that ubiquitylation of PCNA protects stalled forks from extensive DNA2-mediated degradation by ensuring Okazaki fragment maturation and efficient nucleosome reassembly^106^. Consistent with the important functions of histone acetylation in chromatin accessibility and diverse cellular processes including transcription, replication, and DNA repair, PCAF-mediated acetylation of histone H4 Lys8 (H4K8ac) has been shown to engage MRE11 and EXO1 nucleases at stalled forks, resulting in enhanced fork degradation in BRCA1/2-deficient genetic backgrounds^107^. Thus, in response to replication stress, epigenetic modifications of fork histones determine stalled fork stability, and future studies would provide a better understanding of the epigenetic mechanisms of replication fork integrity.

Replication stress leads to genome instability if not resolved in a timely manner by the mechanisms described above. Unresolved replication fork intermediates result in irreversible fork collapse or DSB formation at forks that drive senescence and aging^108^. Other than telomere attrition, replication stress is considered one of the main sources of DNA breaks, which are common features of replicative senescence, oncogene-induced senescence, and aging^108^. Therefore, maintenance of replication fork integrity in response to replication stress is central to preventing genomic instability associated with senescence and accelerated aging.

## Hallmarks of aging driven by replication stress

**Fig. 2:**
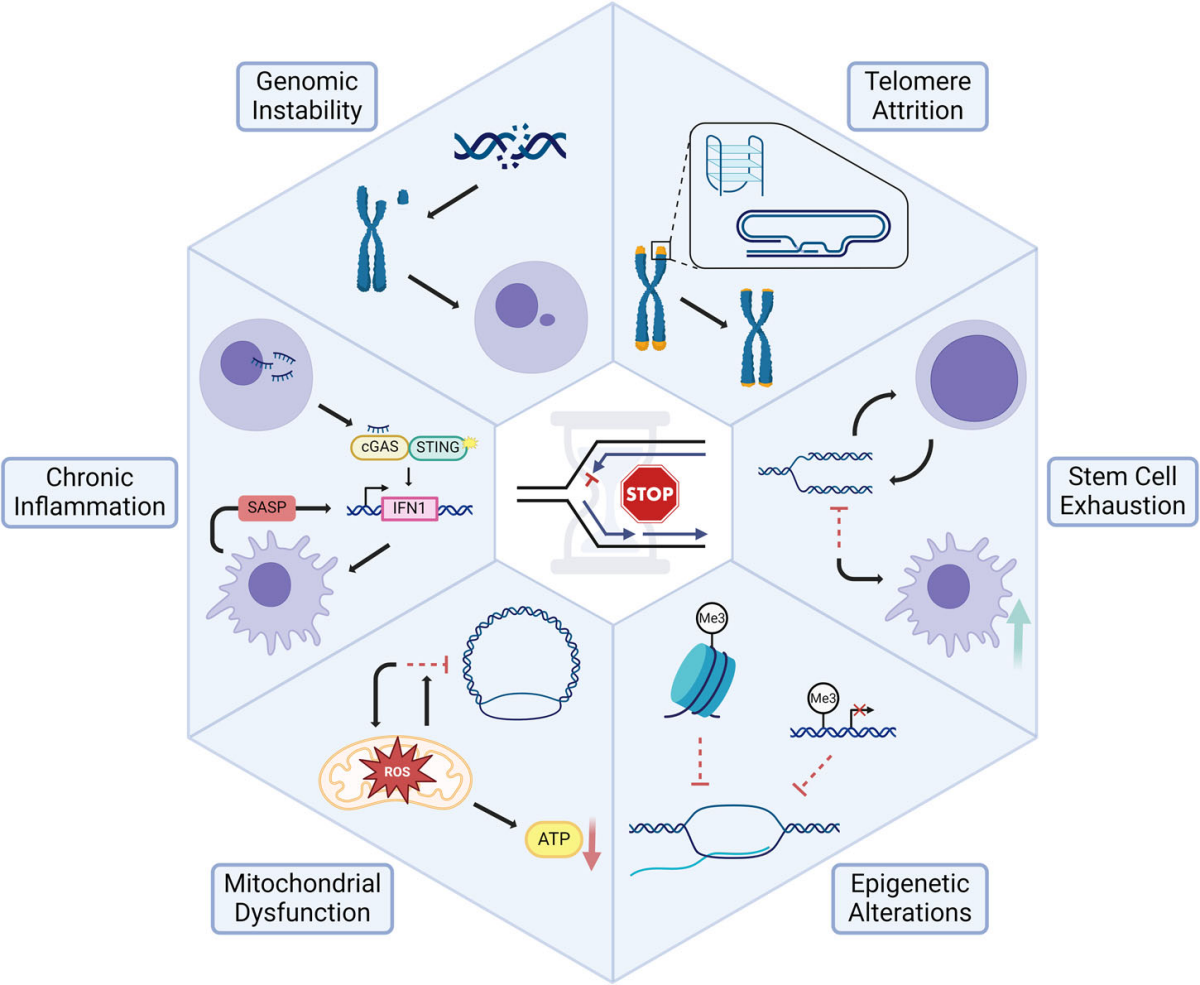
Relationships of replication stress to hallmarks of aging. Evidence from the literature suggests that many of the previously described hallmarks of aging^109^ are driven by replication stress. Representative examples are cited below as well as in the text. *Chronic Inflammation*. Innate immune response activation by LINE-1 element derepression and cytosolic ssDNA incurred by replication stress promotes senescence induction and contributes to sterile inflammation^112,257^. *Genomic Instability*. Replicative stress incurs DNA damage in the form of chromosomal aberrations and DSBs that attenuate replicative lifespan of cells and induce aging-associated tissue dysfunction^9,121,123^. *Telomere Attrition*. Telomeres are fragile sites with DNA sequences prone to form G4 and T-loop secondary structures that break under conditions of replication stress, inducing telomere shortening that limits replicative potential^133,134,139^. *Stem Cell Exhaustion*. Replicative stress in SCs impairs the ability to produce SC progeny. Specific to HSCs, this leads to a variety of tissue-based and organismal dysfunctions including clonal drift and SC attrition^147,151,258^. *Epigenetic Alterations*. Replicative stress recruits a variety of proteins to the stalled replication fork that are involved in remodeling the DNA modification and histone landscapes around the stress. These changes can have long-term effects for cellular senescence^12,259^. *Mitochondrial Dysfunction*. Replication stress in the mitochondria leading to mtDNA mutations causes a deprivation of resources, culminating in premature aging in a pol γ proofreading mutant mouse model^158^. In addition, mtDNA replication errors have consequences for nuclear genome replication and stability, which contribute to aging phenotypes^16^. BioRender was used to create the figure.

Lopez-Otin et al. described nine cardinal hallmarks of aging proposed to be common in multicellular organisms, with a special emphasis placed on aging processes in mammals^5^. The scheme has proven to be highly useful for drawing inferences about the mechanisms underlying aging and framing experimental studies to address their relative importance and how they are interconnected. The field of aging research is not static, and new hallmarks (expanded to 12) emerged with an even greater emphasis placed on robust response to stress being paramount to healthy aging^109^. In the following sections, we discuss selected hallmarks of aging in which replication stress is implicated. Because of their interconnected nature^109^, all hallmarks of aging would experience the impact of replication stress (Fig. 2).

### Chronic inflammation

Loss of replicative potential is an underlying cause of cellular senescence, a hallmark of aging defined as a state of virtually irreversible cell cycle arrest characterized by macromolecular damage coupled with distinct secretory and metabolic profiles^2,109^. LINE-1 retrotransposable elements become transcriptionally derepressed during cellular senescence, reflecting a loss of heterochromatin and leading to activation of the type-I interferon (IFN-1) response characteristic of sterile inflammation^110^. The IFN-1 response induced by cGAS-STING recognition of LINE-1 cDNA contributes to the SASP, a state of inflammatory cytokine, proteinase, and growth modulator secretion that reinforces and promotes senescence induction in proximal cells^2,110^. Chronic inflammation contributes to the age-associated condition of frailty, which has been alleviated in aged mice following treatment with anti-inflammatory agents such as JAK1/2 inhibitors^111^. Inflammatory signaling perpetuated by senescent cell accrual thus contributes to both symptoms and propagation of cellular aging.

Replication stress-induced cytosolic self-DNA accumulation has been demonstrated to effect a senescence-promoting IFN-1 response initiated by cGAS-STING^112^. Cytosolic chromatin fragments in the form of micronuclei trigger the SASP and astrocyte senescence as well as premature neurodegeneration in human AT pluripotent SC-derived brain organoids^112^, exemplifying the role of replication stress in promoting cGAS-STING-mediated senescence induction which contributes to accelerated aging phenotypes.

Depletion of the fork protective factors Abro1 and FANCD2 promotes release of ssDNA fragments from stalled replication forks which leak into the cytosol and activate the cGAS-STING pathway^113^. cGAS-STING-mediated IFN-1 activation is similarly induced by impaired degradation of nascent DNA from stalled replication forks resulting from depletion of the dNTPase SAMHD1, mutated in the inflammatory disorder Aicardi-Goutières Syndrome (AGS)^114^. Moreover, knockdown of the fork protective factors Replication Protein A (RPA) and RAD51 exacerbates IFN-1 signaling in AGS patient fibroblasts that are deficient in TREX1 exonuclease, which functions in degradation of cytosolic single-stranded DNA (ssDNA)^115^. This suggests that replication stress resulting from RPA and RAD51 exhaustion contributes to autoinflammation that promotes senescence phenotypes observed in these cells, including upregulation of interferon-stimulated genes and β-galactosidase positivity^115^.

Cellular senescence and associated aging phenotypes promoted by replication stress are also induced by DNA damage or replicative exhaustion stemming from endogenous or genotoxic sources^2^. Senescence is promoted by a myriad of replication stress-induced processes, including genomic instability, telomere attrition, epigenetic modifications, mitochondrial dysfunction, and SC exhaustion^2^ (discussed below), and the processes of cellular senescence and aging are thus complementary and heavily interrelated in the context of replication stress.

### Genomic instability

Replication stress is a primary source of genomic instability in the form of both chromosomal aberrations and DSBs, which have long been shown to play a causative role in aging^109^. Targeted DSB induction effectuates premature aging phenotypes in murine liver^9^, and systemic mtDNA DSBs induce progeroid phenotypes in rapidly proliferating murine tissues such as thymus and testes^116^. Persistent DSBs have been shown to cause segmental and whole-chromosome aneuploidies in human embryos resulting from chromosomal mis-segregation in mitosis^117^. Heightened chromosome mis-segregation in old-aged human dermal fibroblasts due to an increase in stable kinetochore-microtubule attachments enhances biomarkers of cellular senescence^118^, evidencing the role of chromosomal aberrations in promotion of aging and associated cellular phenotypes.

Nuclear deformation has recently been found to induce replication fork stalling, revealing replication stress as a source of elevated DSB formation observed in cells undergoing mechanical stress^119^. DSB formation is also incurred by replication fork blockage imposed by transcription-replication conflicts, and this DNA damage induction is enhanced in the presence of co-occurring three-stranded DNA-RNA hybrids with the associated non-template ssDNA (R-loops) that exacerbate replication fork stalling^120^. R-loop persistence in mitosis resulting from depletion of the R-loop resolving helicase senataxin (SETX) has also been shown to induce chromosomal fragility and DNA under-replication^121^. Deficiency in the SETX-interacting factor senataxin-associated exonuclease 1 (San1) induces early onset murine cardiomyopathy associated with elevated cardiomyocyte R-loop and DSB levels, evidencing the role of replication stress-induced DNA damage accrual in premature induction of age-related pathologies^122^.

S-phase replication fork stalling, low fork speed, and incomplete DNA replication have been identified as sources of DSB formation and subsequent chromosome breakage that result in segmental aneuploidies in human cleavage-stage embryos^123^. Replication stress incurred by the replicative B-family DNA polymerase inhibitor aphidicolin has been shown to directly stabilize spindle microtubules and induce premature centriole disengagement, causing lagging chromosomes and micronuclei formation^124^. Mitotic segregation defects are also effected by depletion of the structure-specific nucleases ARTEMIS and XPF-ERCC1, and resultant replication fork stalling has been found to increase the incidence of lagging chromosomes and DNA bridges^125^. Deletion of ERCC1 in hematopoietic cells induces immune cell senescence and premature age-related immune dysfunction in mice^126^. Subsequent age-associated tissue damage and senescence marker expression in peripheral organs accompanied by shortened lifespan implicates replication stress-induced DNA damage in promotion of systemic organismal aging.

### Telomere attrition

Telomere instability is an established effector of aging and senescence-inducing replicative exhaustion^109^. Rich in G4 and T-loop secondary structures that impede replication fork progression, telomeres are fragile sites that pose a challenge to replication machinery and are prone to breakage under conditions of endogenous and genotoxin-induced replication stress^127,128^. Telomeric replication fork arrest effectuated by oxidative stress causes aging-associated telomere loss and dysfunction as well as premature cellular senescence^129,130^.

Persistent telomeric secondary structures resulting from RTEL1 helicase deficiency have been shown to cause accrual of reversed replication forks^127^. Reversed forks are aberrantly bound by telomerase, inhibiting replication fork restart and inducing telomere shortening and catastrophe which contribute to pathogenesis of telomere maintenance disorders. Telomere fragility and subsequent senescence induction resulting from replication fork stalling are also effectuated by disruption of RTEL1 helicase binding to the replisome^131^, evidencing the importance of effective replication machinery recruitment to chromosome ends in maintenance of telomere integrity.

Replication and fork protective factors are often localized to telomeres by their association with the shelterin complex, which promotes fork stability and efficient telomeric DNA replication in addition to its general role in telomere end protection^132^. TRF1, the core subunit of the shelterin complex, has been established as a primary facilitator of fork progression by recruitment of the secondary structure-resolving helicases RTEL1, BLM, and WRN, which promote lagging-strand telomere synthesis, as well as topoisomerase IIα (TopoIIα), which is essential for replication intermediate resolution^128,133,134^. Perturbation of TRF1 binding to WRN helicase during S-phase disrupts WRN association with telomeric chromatin, obstructing fork progression and inducing telomere fragility^134^. Depletion of TRF1 similarly destabilizes telomeres by reducing TopoIIα telomere association, impairing resolution of replication-incurred topological stress, and inducing mitotic defects in the form of ultrafine anaphase bridge (UFB) formation^133^. Shelterin subunits TRF1 and TRF2 also associate with the fork protection complex (FPC) subunit Timeless, which preserves telomere length by stabilizing stalled forks and regulating replisome progression through barriers such as the repetitive DNA sequences that constitute telomeres^135^. The FPC subunit Claspin is recruited to telomeres by a specific interaction with TRF2^136^, and overexpression of Claspin and Timeless has been shown to increase cellular replication stress tolerance^137^.

Recent studies have revealed the importance of TRF2 in promoting telomere stability by recruiting origin recognition complex subunit 2 (ORC2), thus promoting formation of telomeric replication origins which rescue compromised replication^138,139^. TRF2 suppresses fork stalling and telomere fragility resulting from transcription-replication conflict by binding the transcription repressor treacle ribosome biogenesis factor 1 (TCOF1) in an S-phase specific interaction^140^. TRF2 has recently been found to promote telomeric fork progression and stalled replication fork restart through S-phase recruitment of Microcephalin 1 (MCPH1), which is implicated in DNA damage signaling and repair and expressed during fetal brain development^141^. Fragile telomeres induced by MCPH1 depletion suggest that telomeric replication stress may contribute to primary microcephaly onset that presents as a clinical symptom of telomere disorder syndromes.

Under conditions of oncogene-induced telomeric DNA replication stress, human cells that lack telomerase activity experience telomere dysfunction-induced senescence (TDIS) characterized by telomere shortening and persistent DNA damage foci accompanied by stable proliferative arrest^142^. Preferential stalling of telomeric replication forks induced by oncogenic signals may suppress malignant cancer progression. Tumor suppression by this oncogene-induced senescence mechanism is mediated by a response to aberrant telomeres in telomerase-negative cells, emphasizing the biological and potential clinical relevance of telomeric replication stress (for a perspective, see ref. ^143^).

### Stem cell exhaustion

The function of an SC is to renew both itself and differentiated progeny throughout an organism’s lifetime^144^. Thus, replication stress occurring within SCs is significantly detrimental to cell populations as well as organismal health. Replication stress in SCs, as such, is a multifaceted issue resulting in clonal drift or complete inability to produce progeny and increased DNA damage, both affecting progeny and DNA damage-associated checkpoints as well as decreasing functionality of specific organismal tissues^145–147^. Deletion of the key replication stress response checkpoint kinase regulator ATR in mice causes a diverse array of age-associated phenotypes, particularly in rapidly proliferating tissues^148^, suggesting that SC exhaustion has disease implications beyond the classic hematopoietic compartment.

Nonetheless, as highly replicative cells, HSCs are a prime example of the consequences of replicative stress. HSCs confronting replication stress and associated cellular damage experience high levels of attrition, contributing to the pathology of the bone marrow failure disorder FA^147,149^. The decline of HSCs accompanies global tissue degeneration and malignant transformation in cancer patients^147^. With a lack of blood cell production, tissues atrophy due to decreased oxygen supply, become susceptible to infection, and exhibit a reduced ability to protect against endogenous or exogenously induced DNA damage^150,151^.

Once DNA damage arises from dysfunctional replication, a series of checkpoints are activated to deal with the cellular stress that often detriment the function of SCs, notably in the hematopoietic compartment^151^. Upon checkpoint activation, a decline in SC proliferation occurs followed closely by a SASP-mediated inflammatory response and clonal drift. This often leads to selection of malignant clones and impaired tissue maintenance. Moreover, chronic inflammation compromises the immune system and others (e.g., cardiovascular)^152^. On a larger physiological basis and from the perspective of aging-related phenotypes, SC attrition can cause a myriad of different premature aging disorders and related symptoms. While replicative stress in HSCs is often considered in the context of FA, SC replicative stress in general is associated with muscle atrophy, impaired immune response, metabolic issues such as obesity and diabetes, inflammation, osteoporosis, WS-related cardiac decline, and other cardinal features of aging^8,153^.

While SC attrition and clonal drift induced by replicative stress are evidenced in many tissues, these defective processes may arise by a related mechanism^147,151^. For example, the same cellular issues that are responsible for HSC attrition and contribute to FA are also implicated in inflammatory responses and the SASP^147,151^. As such, SC replicative stress may be a good therapeutic target to mitigate aging-related pathologies, counteracting activated checkpoints, the inflammatory response, or negative selection of affected SCs. This would most likely be accomplished by inhibition of HSC-associated senescence but may lead to elevated cancer risk.

Reprogramming of somatic cells into induced pluripotent stem cells (iPSCs) has attracted interest in biomedical therapies. However, iPSCs are frequently characterized by genomic instability and elevated DNA damage, which may compromise their utility for replenishment treatments. Ironically, the rejuvenating Yamanaka factors (OCT4, SOX2, KLF4, cMYC) cause replication stress which in turn activates the DNA damage response^154^. Strategies such as CHK1 augmentation or nucleoside supplementation to limit replication stress during somatic cell reprogramming were shown to reduce genomic instability in iPSCs^154^, which may increase their efficacy for safe use in clinical therapies.

### Epigenetic alterations

Replicative stress is not known to alter the epigenetic landscape in the vicinity of a stalled replication fork. Rather, the DNA damage response that is activated to alleviate replication stress can alter the epigenetic landscape and cause drastic long-term effects on the cellular and potentially organismal levels^155^. This occurs both as DNA-level modifications as well as changes to the histones in the vicinity of the stalled fork.

One of the initial steps in the replication stress response is recognition and binding of exposed ssDNA in the vicinity of the stalled fork by RPA. Subsequently, RPA interacts with the histone cell cycle regulator complex (HIRA) to deposit histone H3.3 and H4 proximal to promoters, enhancers, and other gene elements to modulate transcription of associated gene bodies^155^. When extensive fork stalling enables RPA to remain bound to ssDNA and form a HIRA complex for a prolonged period of time, histone placement and modification become varied, changing how those regions are normally transcribed^155^. In addition to RPA, other factors such as Atrx and Daxx associate with H3.3 at telomeres to repress telomeric RNA production, suggesting that both replication stress and factors associated with specific genomic loci play a role in H3.3 localization and resultant transcriptional regulation.

HIRA and another chromatin-regulating factor known as ASF1a (with which HIRA physically interacts) were found to play a role in the formation of senescence-associated heterochromatin foci (SAHF)^156^. During cellular senescence, ASF1a and HIRA act to promote assembly of proliferation-promoting genes into chromatin which discourages resident gene expression; moreover, HIRA-ASF1a complex-induced formation of SAHF provides the opportunity for cell cycle exit during senescence. SAHF are implicated in oncogene-induced replication stress and their formation is dependent on the DNA replication stress response checkpoint kinase ATR^157^. Thus, SAHF may serve as a valuable biomarker of oncogene-driven replication stress, signifying heterochromatin perturbation which in turn harnesses DNA damage response signaling. Epigenetic manipulation of the heterochromatin state in vivo to relax it with a histone deacetylase inhibitor enhanced the DNA damage response and triggered tumor regression^157^. This work supports efforts to pharmacologically modulate heterochromatin to treat tumors by targeting the DNA damage response.

Recently, DSBs have been evidenced to induce epigenetic modifications that are associated with aging-related phenotypes^12^. Specifically, histone H3 and H4 modification and rearrangement, causing differing levels of methylation and acetylation, decrease Waddington Valley differentiation and were shown to accelerate aging in mice^12^. It is likely that replication stress-induced DSB formation is largely responsible for this innate change in histone modifications. It is plausible that modification of histone H3.3 and H4 by the RPA-HIRA-histone H3.3 complex causes transcriptional changes associated with the same phenotypic changes evidenced in the study by ref. ^12^.

Interestingly, the organismal and phenotypic characteristics attributed to epigenetic changes are some of the most strongly correlated with aging and cellular degeneration. Yang et al. showed that DSB induction resulting in epigenetic rearrangement induced cellular and behavioral phenotypes of neural degeneration, including reduced memory^12^. Additionally, they demonstrated muscle atrophy, lack and discoloration of hair, skin abnormalities, and other phenotypes that closely mirror the pathology of advanced aging^12^. It is plausible that replicative stress that causes DNA breaks and histone rearrangements induces similar epigenetic rearrangements, thereby indirectly leading to an acceleration of the aging phenotype.

### Mitochondrial dysfunction

A hugely consequential but often underappreciated effect of mitochondrial replicative stress on aging was evidenced by observations of mice genetically deficient in the proofreading subunit of the nuclear-encoded replicative DNA polymerase gamma (pol γ) that is responsible for replication of the mitochondrial genome^158^. The 3- to 5-fold elevated point mutations and increased deletions in the mitochondrial genome of pol γ mutant mice with a defective proofreading subunit cause reduced lifespan and accelerated aging phenotypes including reduced weight and subcutaneous fat, hair loss, spine curvature, osteoporosis, blood anemia, oversized heart, and compromised fertility. These symptoms first started to appear when the mice were ~25 weeks old, which is considered young adulthood. This was a major finding in gerontology research as it provided evidence to support the theory that mutation burden in the mitochondrial genome due to defective proofreading during replication may be a driving force of aging.

With this new advance, the field was intrigued by the possibility that mitochondrial mutation held rank over accumulation of mitochondrial reactive oxygen species (ROS) as the primary driver of aging. Indeed, Trifunovic et al. found that the level of ROS in the pol γ mutator mice was normal, and the mouse embryonic fibroblasts (MEFs) from these mice did not show a hypersensitivity to oxidative stress^159^. Collectively, the authors suggested that respiratory chain defects are not the driver of premature aging in the mtDNA mutator mice. Consistent with this conclusion, Vermulst et al. found that the pol γ mutator mice accumulated mtDNA deletions at an elevated rate with age in brain and heart tissue^160^. Although it is tempting to speculate that premature aging of the pol γ mutator mouse is attributed to error-prone replication, Martin and Loeb point out that it is dubious to discount the oxidative theory of aging as a driver of the mutator mouse phenotypes because the mutations in respiratory-chain proteins of the pol γ mutant mitochondria would result in elevated ROS and a leaky proton gradient^161^, representing a causative factor for the premature aging features.

Crosstalk between mitochondrial and nuclear genome metabolism is thought to play a role in aging, but the devil lies in the details. Using the pol γ proofreading mutant mouse model described above, Hamalainen et al. found that cells from the mutator mice displayed compromised nuclear DNA replication and accumulation of DSBs, especially in the SC compartment^16^. It was proposed that mitochondrial replication stress and mutation of the organelle’s genome deprive the nuclear genome of resources as the cell attempts to remedy the induced stress. Once mitochondrial replication stress occurs, replicative resources are sequestered in the mitochondria, causing an overall decrease in cellular nucleotide and deoxynucleoside triphosphate (dNTP) pools, therefore affecting nuclear genome stability and ability to replicate^16^. However, in a recent study, Sharma reported that genetic mtDNA polymerase γ proofreading deficiency in mouse embryos did not perturb total dNTP pools as demonstrated by a technique involving the HPLC/UV-detection approach, which is contended by the authors to produce highly reliable results^162^. Sharma et al. concluded that alterations in dNTP pools are likely not the underlying cause of premature aging in the pol γ proofreading mutant mouse model. Further studies are necessary. Despite this caveat, the central role of mitochondria in the cell suggests that when mitochondrial-encoded proteins are dysfunctional due to errors in mtDNA replication, other hallmarks of aging are likely to be affected. From depletion of cellular resources, increased ROS production, and decreased phosphate-rich energy production, mitochondrial replication stress likely directly contributes to age-related frailty; however, therapeutics targeting mitochondrial replication stress have not been widely developed to date.

## Replicative stress as a biomarker or cause of cellular senescence and accelerated aging: evidence from translational models, genetic diseases, and clinical studies

There is much research supporting the theme that DNA damage universally causes organismal decline via its effect on multiple pillars of aging^65^. However, experimental evidence (as discussed above) demonstrates that replication stress causes genomic instability and endogenous DNA damage which also promotes aging and age-related diseases mediated in large part by advancing cellular senescence. Thus, aging researchers are very interested in genomic-based biomarkers for cellular senescence^64,163^. Many of these are based on DNA damage and the DNA damage response. Examples include γ-H2AX, MRE11, RAD50, NBS1, ATM, ATR, 53BP1, MDC1, RAD17, and telomere dysfunction-induced foci (TIF)^164^. In addition, a reduced level of DNA synthesis in cells or tissues detected by fluorescent staining of nucleoside analogs such as bromodeoxyuridine (BrdU) and ethynyl-2’-deoxyuridine (EdU) incorporated into genomic DNA serves as a reliable biomarker of replication stress in senescent cells. Cell cycle arrest-based markers align well with biomarkers of replication stress in senescent cells. Other biomarkers of cellular senescence such as the senescence-associated lysosomal enzyme β-galactosidase, epigenetic modifications, and the SASP are associated with replication stress. The distinction between biomarkers of the DNA damage response versus replication stress response may be in part blurred due to their overlapping nature. In fact, some of the classic DSB repair proteins such as MRE11 are implicated in stalled replication fork processing^95^. Many factors involved in replication fork reversal triggered by fork stalling are also involved in DSB repair^165^, making it difficult to strictly assign them as biomarkers of replication stress versus the DSB.

In the following sections, we will consider the experimental evidence and conceptual logic for replication stress as a driver of cellular senescence and aging and apply it to existing models and emerging topics that are relevant in translational and clinical realms. Characterizing how replication stress directly or indirectly drives phenotypes of accelerated aging will set the stage for developing a new understanding of the factors and conditions that might be modulated in replicative tissues to modulate debilitating mechanisms of cellular senescence and potentially ameliorate age-related disease pathways.

### Replicative senescence as a biomarker for aging

The cyclin-dependent kinase inhibitor and tumor suppressor p16^INK4a^ has been useful in studying both neoplastic transformation during carcinogenesis and development of aging phenotypes in mouse models^166^. Expression of p16^INK4a^ is significantly elevated during cellular senescence, prompting researchers to consider the signaling molecule as a potential biomarker for aging in humans as well (reviewed in ref. ^167^). Taking note that the transcription factor CUX1 regulates expression of p16^INK4a^ and other tumor suppressor genes, Jiang et al. determined that endothelial cells in passage 10 compared to passage 5 displayed greater CUX1 and p16^INK4a^ expression accompanied by elevated γ-H2AX and decreased telomere length, suggesting replicative senescence^168^. Conversely, depletion of CUX1 in late passage endothelial cells caused reduced p16^INK4a^ expression, reduced β-galactosidase and γ-H2AX staining, restored BrdU incorporation indicative of more normal DNA replication, and ameliorated cell cycle arrest, all parameters pointing toward a role of CUX1 and p16^INK4a^ in regulating replicative senescence. Similar findings were reported for human primary vascular smooth muscle cells, suggesting that the effects were not specific to endothelial cells.

### Lifespan extension of ATR mutant mice by increased Rrm2 gene dosage

The central signaling axis for replication stress in mammals is mediated by the ATR kinase and its downstream target kinase CHK1. It follows that ATR or CHK1 may serve as a biomarker or even a therapeutic target for aging. Lopez-Contreras et al. explored the hypothesis that premature aging of transgenic ATR mutant mice could be suppressed by increased dosage of the *Rrm2* gene, which encodes a regulatory subunit RRM2 of ribonucleotide reductase implicated in the production of dNTPs required for replicative DNA synthesis^169^, building on findings that replication stress is reduced by nucleoside addition^170,171^. Elevated *Rrm2* gene dosage indeed prolonged survival of ATR mutant mice and depressed chromosomal breakage at fragile sites^169^. However, lifespan of wild-type laboratory mice was not extended by increased gene dosage of *Rrm2* and/or *Chk1*, leading the authors to suggest that protection from replication stress using the transgenic mouse model system employed did not extend normal aging^172^.

### Aging hematopoietic stem cells driven to the brink by replicative stress

A key advance fueling the theme that replicative stress drives aging was put forth by Flach et al. in 2014 with their *Nature* paper^8^ showing that cycling aged HSCs that display replication defects and reduced expression of the replicative DNA helicase MCM have compromised ribosomal (r) DNA gene status characterized by elevated γ-H2AX due to a mis-localized phosphatase, notably *without* the expected DNA damage response activation or a detectable level of DNA DSBs. The quiescent old HSCs display compromised self-renewal, a biased myeloid differentiation signal, transcriptional silencing, and reduced ribosome biogenesis, suggesting a mechanism of aging that is independent of DNA damage or DNA damage response activation and relies heavily upon replication stress as the driver of declined functional output. Epigenetic histone modifications, as suggested by the γ-H2AX enrichment, may drive changes in expression of replication machinery proteins (like the MCM helicase) as well as down-regulate rDNA transcription. Whether these changes can be remedied therapeutically in bone marrow failure syndromes remains to be seen. Replication stress may not be the only driver of aging for HSCs, as suggested by a recent study showing that genotoxic aldehyde which causes DNA damage drives aging in a p53-dependent manner^41^. Even these latest findings, however, do not discount the importance of replication stress in the aging pathway as metabolism-derived formaldehyde-induced DNA damage is likely to perturb fork progression as well, as suggested by studies that examined the role of the fork protection factors BRCA2 and FANCD2 in cells experiencing endogenous aldehyde toxicity^173^.

DNA damage tolerance (DDT) is an important pathway for dealing with stalled replication forks. Pilzecker et al. investigated the importance of DDT in HSCs and multipotent progenitors using a mouse Pcna^*K164R/K164R*^ model engineered to be defective in transitioning from a replicative mode to a DNA damage-tolerant mode of replication^174^. By studying the bone marrow of DDT-deficient Pcna^*K164R/K164R*^ mice, the researchers determined that the hematopoietic compartment displayed accelerated aging, indicative that the DDT response to replication stress in the murine model is critical for long-term HSC fitness and tissue homeostasis.

The role of replicative stress in aging of SCs other than those of the hematopoietic compartment remains to be fully characterized. However, disease models characterized by accelerated aging and elevated DNA damage in SCs have been described (for perspective, see ref. ^175^). As discussed by Goodell and Rando^7^, a myriad of factors revolving around genetic mutations, epigenetic changes, and environmental milieu play a role in SC functionality over the lifetime of an individual. It is reasonable to postulate that depletion of the SC reservoir by replication stress promotes decline of tissues and organs in a cell lineage-dependent manner. Although there was not a measurable level of DNA breaks detected in the aging HSCs described by ref. ^8^, in certain contexts inducible DSBs contribute to cellular senescence and aging (see below).

### Double-strand breaks that may arise during replication stress cause accelerated aging

As detailed in previous sections, cellular senescence driven by replication defects is considered a hallmark of aging. This prompts one to consider replicative stress as a potentially useful biomarker for aging. Although cell metabolism markers such as β-galactosidase staining have been a popular marker for senescent cells^176^, markers of replication stress have not been as extensively studied. Rather, DNA damage emanating from replication stress or by other avenues (e.g., oxidative stress^177^) has been postulated as a key biomarker for cellular senescence and even organismal aging. One of the most prominent DNA lesions associated with changes to the genome that is implicated in (and perhaps a driving force of) aging is the DSB^10^, one of the most lethal forms of DNA damage and a source of great genomic instability due to its recombinogenic nature. A signature paper from the Vijg lab provided evidence that inducible DSBs cause accelerated aging of mouse liver^9^.

Recently, the Sinclair lab and collaborators developed an inducible DNA break mouse model that enabled them to investigate the importance of epigenetic changes induced by chromosome breaks for aging^12^. Alterations in epigenetic landscape in regions surrounding the DSBs were associated with aging phenotypes at the cellular and organismal levels. However, whether the aging phenotypes associated with epigenetic changes are reversible at the organismal level remains to be seen (for a perspective, see ref. ^178^). Nonetheless, the described model system will be useful for future work to study in vitro and in vivo aging. It remains to be determined if DSBs deriving from replication stress drive aging in replicative tissues by a mechanism that is different from the one described above, in which DSBs introduced frankly by the in vivo inducible restriction endonuclease system in both non-replicative and replicative tissues cause aging in a manner that is heavily dependent on epigenetic changes.

Although one could argue that DSBs represent only one of multiple DNA lesions to induce accelerated aging, the probability that they occur at the fork in replicative tissues in vivo is high. Replication fork stalling followed by blockage leads to single-stranded and ultimately DSBs, i.e., broken replication forks that cells must deal with using fork reconstruction pathways to preserve genomic stability (*see Replication Fork Stalling Section*). Typically, these repair mechanisms to heal DSBs at broken replication forks involve HR repair or the less faithful nonhomologous end-joining (NHEJ). Although stalled forks can be restarted by non-recombinogenic mechanisms, the transient ssDNA that arises is susceptible to breakage. Thus, it is difficult to tease out if a structural feature of the stalled or arrested replication fork, the fork-associated DSB itself, or both represent a key signaling event in cellular senescence and aging. Either way, in proliferating cells of rapidly turning over tissues, replication stress is a driving force for age-associated signaling pathways associated with delayed fork progression.

### Chemotherapeutic senescence-causing drugs that interfere with replication fork progression

Therapy-induced senescence achieved by application of chemotherapeutic DNA damaging agents is a front-line strategy to combat cancer^179^. However, cancer survivors having undergone chemotherapy often display premature aging due to DNA damage by distinguishing underlying mechanisms, including the consequence of perturbing replication fork integrity^180^.

Common examples of chemotherapy drugs that not only induce caustic DNA damage but also deter replication fork progression include DNA cross-linking agents (cisplatin, methotrexate) and drugs that induce bulky DNA lesions (e.g., alkylating agents such as temozolomide, cyclophosphamide, etc.). However, even beyond such drugs that directly induce DNA damage are agents such as etoposide, mitoxantrone, camptothecin, irinotecan, topotecan, and derivatives that poison topoisomerases which relieve torsional stress at the fork to ensure smooth fork progression. Antimetabolites, including gemcitabine, hydroxyurea, and paclitaxel disturb the normal nucleotide pool, causing a reduction in replication fork rate. Even certain poly(ADP) ribose polymerase (PARP) inhibitors which were historically thought to solely exert their action by interfering with PARP’s role in the repair of DNA strand breaks are now also believed to exert cytotoxicity against cancer cells by trapping PARP on genomic DNA^181^, potentially interfering with cellular DNA replication progression and other nucleic acid metabolizing events.

Interestingly, certain topoisomerase inhibitors act by making toxic covalent DNA-topoisomerase protein complexes^182^, approximating the tight (but noncovalent) PARP-DNA complexes. In a broader sense, it is of growing interest to ascertain the role of DPCs in the mechanism of action for certain chemotherapy drugs and cellular proteases (e.g., SPRTN^183^) that act to degrade the DNA-interacting protein. Presumably, DPCs are a source of replication stress. As noted earlier, a hereditary premature aging disorder known as Ruijs-Aalfs syndrome is characterized by mutant alleles of the SPRTN protease implicated in DPC digestion^53,54^ (Table 2). While studies suggested SPRTN acts as a DPC protease specifically at replication forks^184,185^, a recent work provided evidence that SPRTN patient variants are defective in global genome DPC cleavage^183^. It will be of great interest to ascertain if SPRTN and other factors implicated in DPC repair (e.g., FANCJ helicase mutated in FA^186^) mediate functions in replication stress paramount to the suppression of aging phenotypes.

All of the scenarios mentioned above pose a challenge to cancer biologists to comprehend the dominant mechanism of action of senescence-causing drugs. What was once thought to be classic DNA damage therapy now entails replicative stress as a major component of not only the anti-cancer properties but also the potential age-associated symptoms experienced by many cancer patients undergoing chemotherapy treatments.

### Senolytic drugs targeting replicative senescent cells to combat aging

As expertly summarized in recent reviews^4,187^, an opportunistic approach to extending health span is to target senescent cell viability with a new and emerging class of compounds known as senolytic drugs. The principle is simple: elimination of senescent cells by senolytic drugs would suppress the SASP, which is directly implicated in aging and age-related diseases. Alternatively, senomorphic drugs act to suppress cell-extrinsic effects of senescent cells (rather than eliminate senescent cells altogether); a good example is the SASP inhibitors. Senotherapeutics are being used to target adipose tissue, bone, skeletal muscle, brain, liver, and the cardiovascular system.

While DNA damage-induced senescent cells that elicit inflammatory pathways due to accumulation of cytosolic nucleic acids are frequently targeted by senotherapeutic drugs, the question arises: Can cells characterized by elevated replication stress be an optimal target of senolytic drugs? In other words, can senescence be prevented by targeting the heightened replication stress that may precede the DNA damage? Or does replication stress represent a biomarker for senolytic drugs? The fact that DNA damage drives cellular senescence prompts consideration of potential therapeutic strategies that exploit genetic and environmental factors to selectively eliminate cells that age prematurely (senescence) due to aberrant fork progression leading to DNA breaks, thereby keeping the level of SASP under control. It is plausible that characteristics of replicative stress might serve as novel biomarkers for senescence upstream of many of the cellular properties used to detect senescence. The activated signaling pathways (e.g., chemokines, interleukins, inflammatory factors, proteases, etc.) may ultimately derive from compromised replicative synthesis which may serve as a direct signal or be transmitted to the cellular machinery via DNA damage such as single-stranded and double-stranded DNA breaks.

Drug screening to identify classes of senolytic drugs that act to target cells that display compromised replicative DNA synthesis may prove to be informative and valuable, as replicative stress conceivably precedes genomic DNA damage in many cases. The targeted mechanism of action for drugs that target cells experiencing replication stress would well precede cell signaling pathways that may be less efficacious or pose undesired drug cytotoxicity toward normal cells.

From a more clinical viewpoint, collective evidence demonstrates that senescent cells that become enriched with age are found to be highly abundant at actual diseased sites (tissues, organs)^188^. The idea that senescence drives aging and age-related diseases in a broad spectrum of tissues including adipose, bone, skeletal muscle, brain, cardiovascular system, and liver is nicely summarized in a 2021 review by Robbins et al.^4^. A working model has arisen that reduction of senescent cell viability via senolytic drugs that kill senescent cells or senomorphic/senostatic drugs that suppress disease-causing phenotypes of senescent cells offers mechanistic approaches to extend health span. With many new and ongoing clinical trials for senotherapeutics underway, it is prudent to carefully study their mechanisms of action to ascertain if classes of drugs preferentially kill cells experiencing replication stress-induced senescence.

## Summary

Replication stress has emerged as a central focus in eukaryotic chromosome biology and a driving force in hereditary diseases characterized by genomic instability and features of accelerated aging. Building upon these seminal human genetic studies, molecular biologists and nucleic acid biochemists have discovered many of the molecular mechanisms underlying replication stress that not only compromise the speed of DNA synthesis but also assault replication fork integrity. Both endogenously folded DNA structures such as G4s as well as exogenously induced DNA damage caused by environmental stress can induce replication stress; moreover, genetic deficiency of fork protection factors exacerbates the destabilization of replication forks. Combined, chemically induced and genetic synthetic lethality may result.

**Fig. 3:**
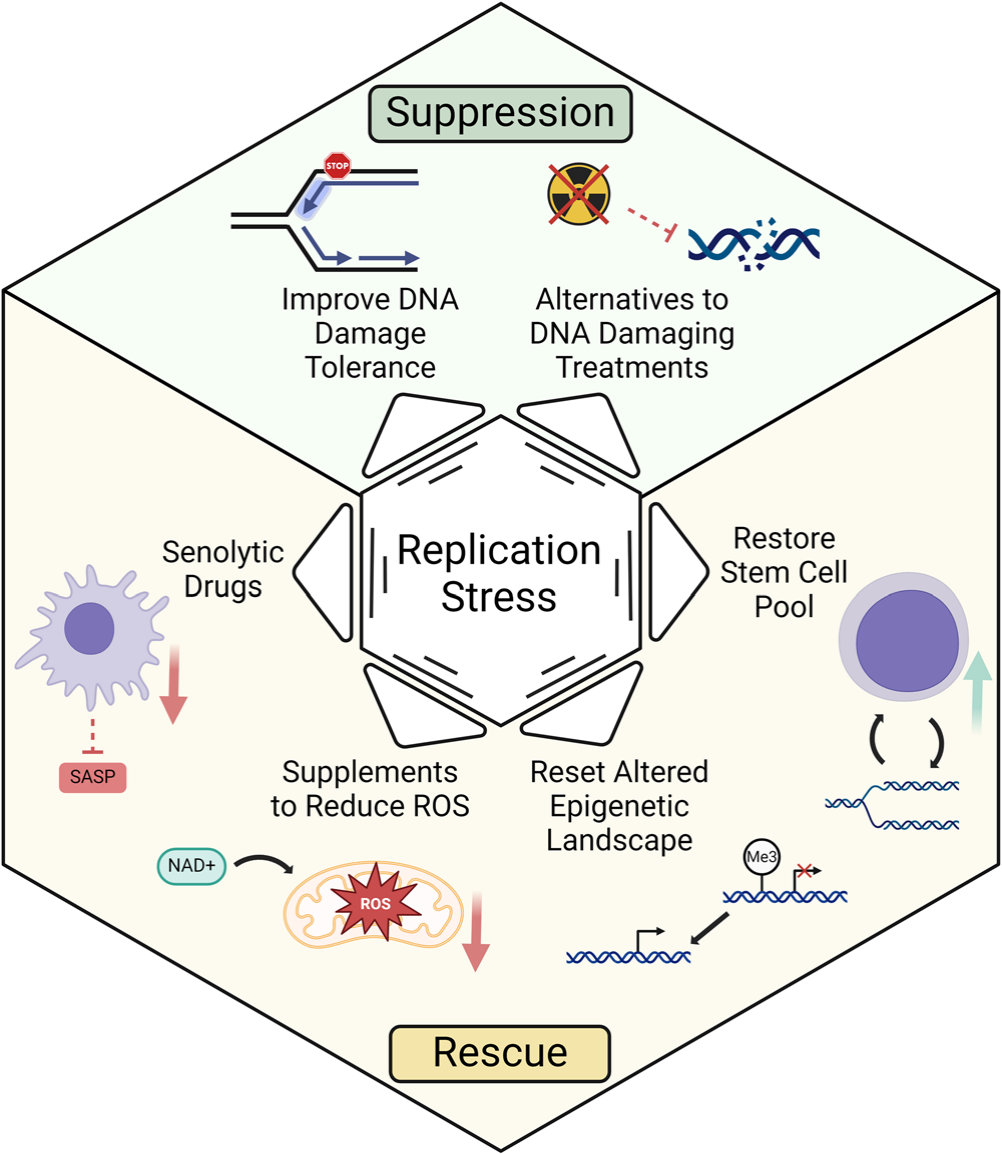
Avenues to suppress replication stress or rescue the aberrant phenotypes caused by replication stress. The upper third of the hexagon depicts two mechanisms to avoid replication stress. The lower two-thirds of the hexagon depicts four strategies that alleviate the consequences of replication stress. See text for details. BioRender was used to create the figure.

There is great interest in mechanisms whereby replication stress is suppressed or the outcomes induced by replication stress can be rescued (Fig. 3), as discussed in this review. A key DNA insult arising from replication stress is the DSB, which has been shown to cause epigenetic changes and drive cellular senescence and aging in elegant biological systems; resetting altered epigenetic landscapes caused by DNA damage to combat aging is gaining support. In addition to hereditary premature aging disorders, replication stress can also cause organ decline associated with normal aging.

As mentioned above, chemotherapeutic senescence-causing drugs or radiation exert negative cytotoxic side effects for normal cells and tissues, prompting new avenues of basic science and clinical research. For example, there is discussion of whether circadian clock genes can act to suppress tumors and if chronochemotherapy can be optimized^189^. Cancer cells have acquired mechanisms to withstand chemotherapy drug-induced replication stress, and transcriptional programs are proposed to serve as biomarkers and potential targets^190^. Combinations of chemotherapy drugs and immunotherapies have risen in the clinic^191^.

Replicative senescence has emerged as a biomarker for aging and age-related diseases. An ever-expanding body of evidence suggests that replication stress is the driving element in SC decline, particularly in the hematopoietic compartment. Thus, restoring the SC pool remains a viable option for some genetic diseases. As discussed on the Fanconi Anemia Research Fund (FARF) website, allogeneic HSC transplantation is the one durable cure for blood deficiencies in FA (Treatment | Fanconi Anemia Research Fund).

On the new frontier to promote healthy aging lie senolytic drugs that eliminate replicative senescent cells altogether. As the scientific and lay communities were poised to celebrate DNA repair in the 1990’s^192^ (Contents | Science 266, 5193), the 21st century brings robust interest in the replication stress response anticipated to be accompanied by new advances in medicine to improve cancer and aging outcomes.
